# Modeling pandemic to endemic patterns of SARS-CoV-2 transmission using parameters estimated from animal model data

**DOI:** 10.1093/pnasnexus/pgac096

**Published:** 2022-07-01

**Authors:** Sarah Mullin, Brent Vander Wyk, Jennifer L Asher, Susan R Compton, Heather G Allore, Caroline J Zeiss

**Affiliations:** Yale Center for Medical Informatics, Yale School of Medicine, New Haven, CT 06520, USA; Department of Internal Medicine, Yale School of Medicine, New Haven, CT 06520, USA; Department of Comparative Medicine, Yale School of Medicine, New Haven, CT 06520, USA; Department of Comparative Medicine, Yale School of Medicine, New Haven, CT 06520, USA; Department of Internal Medicine, Yale School of Medicine, New Haven, CT 06520, USA; Department of Biostatistics, Yale School of Public Health, New Haven, CT 06520, USA; Department of Comparative Medicine, Yale School of Medicine, New Haven, CT 06520, USA

**Keywords:** models, translational, infection, endemic, SARS-CoV-2

## Abstract

The contours of endemic coronaviral disease in humans and other animals are shaped by the tendency of coronaviruses to generate new variants superimposed upon nonsterilizing immunity. Consequently, patterns of coronaviral reinfection in animals can inform the emerging endemic state of the SARS-CoV-2 pandemic. We generated controlled reinfection data after high and low risk natural exposure or heterologous vaccination to sialodacryoadenitis virus (SDAV) in rats. Using deterministic compartmental models, we utilized in vivo estimates from these experiments to model the combined effects of variable transmission rates, variable duration of immunity, successive waves of variants, and vaccination on patterns of viral transmission. Using rat experiment-derived estimates, an endemic state achieved by natural infection alone occurred after a median of 724 days with approximately 41.3% of the population susceptible to reinfection. After accounting for translationally altered parameters between rat-derived data and human SARS-CoV-2 transmission, and after introducing vaccination, we arrived at a median time to endemic stability of 1437 (IQR = 749.25) days with a median 15.4% of the population remaining susceptible. We extended the models to introduce successive variants with increasing transmissibility and included the effect of varying duration of immunity. As seen with endemic coronaviral infections in other animals, transmission states are altered by introduction of new variants, even with vaccination. However, vaccination combined with natural immunity maintains a lower prevalence of infection than natural infection alone and provides greater resilience against the effects of transmissible variants.

Significance Statement The pandemic to endemic trajectory of SARS-CoV-2 transmission will be shaped by the tendency of coronaviruses to elicit nonsterilizing immunity and generate new variants. We utilized estimates from controlled rat coronaviral infection in deterministic compartmental models to inform routes to endemic stability in SARS-CoV-2. We introduced translationally altered parameters to explore the effects of waning immunity, exposure to increasingly transmissible variants, and successive vaccination. We arrived at an endemic state in which 15% of the population remains susceptible to reinfection. Similar to endemic coronaviral infections in other animals, transmission states are altered by introduction of new variants, even with vaccination. Accumulating and maintaining evolving immunity through vaccination and inevitable natural exposure is essential to achieving a stable endemic state.

## Introduction

Predicting the path of COVID-19 to endemic status can be aided by study of other endemic animal coronaviruses (1–6). Common elements of coronaviral infection in humans and animals is their tendency to cross the species barrier (7, 8), and to follow a pandemic-to-endemic trajectory shaped by nonsterilizing immunity (4, 5, 9), generally declining disease severity (9, 10), immune evasion following vaccination (3, 11), and periodic resurgence associated with new variants (6, 12, 13).

In this paper, we develop compartmental models of COVID-19 pandemic to endemic transition using parameter estimates derived from controlled coronaviral infection study in rats (14). Sialodacryoadenitis virus (SDAV) is a highly infectious betacoronavirus (2, 9, 15) that causes transient respiratory disease in rats (16, 17). SDAV is closely related to two human upper respiratory pathogens, human coronavirus HKU1 (HCoV-HKU1) and human coronavirus OC43 (HCoV-OC43) (8, 18, 19). Like human immunity to seasonal coronavirus (20) and SARS-CoV-2 natural infection (21) or vaccination (22–24), immunity in rats elicited by SDAV infection (5, 25, 26) or heterologous coronaviral vaccination (25) is temporary.

SARS-CoV-2 transmission dynamic models have been published and informed policies on mitigation strategies, vaccination roll-out, and healthcare settings (27–29). Some models, both deterministic and stochastic, explore waning immunity with the susceptible population receiving those who have lost their immunity over time (30–32). However, due to the observational nature of these epidemiological models, the resulting mathematical models may not yield accurate insights of disease dynamics (33). Using SDAV in rats, we generated controlled infection and reinfection data after high and low risk viral exposures (34) or vaccination with a heterologous virus (25). Deterministic compartmental models explored pandemic-to-endemic transition using rat-derived in vivo transmission estimates as a starting point. This was followed by the introduction of translationally altered parameters based on human SARS-CoV-2 transmission to explore the effects of waning immunity, exposure to variants with increased transmissibility, and successive vaccination.

## Results

### Model 1: naturally acquired coronaviral transmission prior to vaccination

**Fig. 1.**
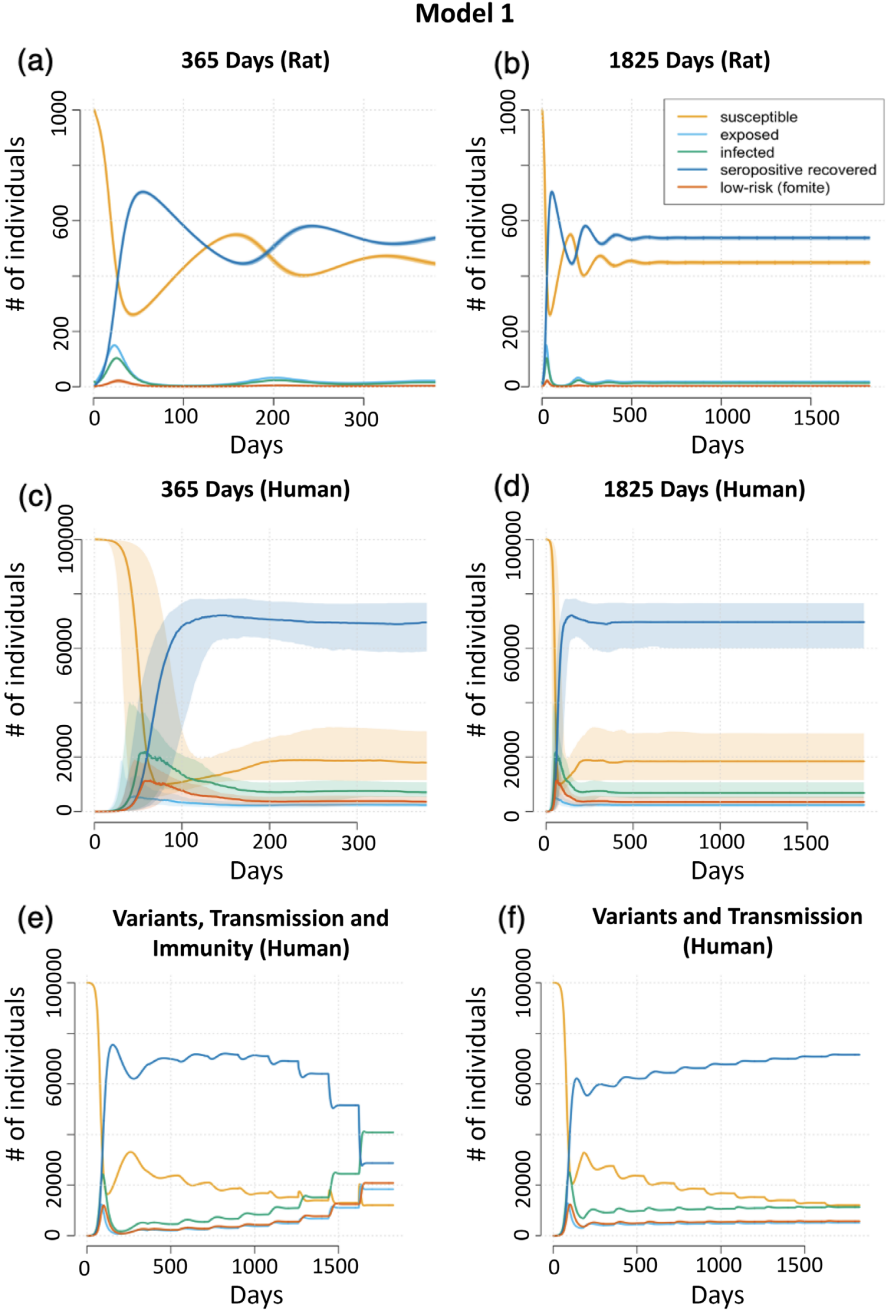
SARS-CoV-2 Disease Compartment Curves for Model 1 with predominantly in vivo rat study estimates (a) and (b) and human-adapted estimates (c) and (d) accounting for the effects of successive variants and varying immunity (e) and (f). (a) and (b): using predominantly rat data, iterated for 365 days (a) and for 1,825 days (b). The model stabilizes after approximately 724 days at an endemic state with approximately 41% of the population susceptible to reinfection. (c) and (d): using translational human estimates, iterated for 365 days (c) and for 1,825 days (d). Curves are the median at time *t* (25th and 75th percentile) to show the sensitivity of the curves to our set of chosen parameters. When an analysis of endemic stability across all parameter choices is performed (Figure S4, Supplementary Material), endemic stability is reached at a median of 1,119 days (IQR = 1017.75). (e) and (f): assessing an introduction of variants into our SARS-CoV-2 Disease Compartment Curves with human estimates and introducing increasingly transmissible variants at 180-day intervals. In (e), we model decreasing length of natural immune protection from reinfection, whereas in (f), immune protection is held constant. The compartments and associated curves are denoted in the legend and are defined as follows: susceptible (yellow; S), exposed (light blue; E), infected (green; I), seropositive recovered (dark blue; *R*^+^), and low-risk/fomite (orange; L). Population size (closed with no births or deaths) is given as # of individuals on the *Y-*axis.

The compartmental curves using estimates obtained from rat (Table 1) and human adapted data (Table 2) are shown in Figure1 with time iterations from 1 year (365 days) and 5 years (1,825 days). Translational considerations regarding differences in transmission of SDAV in rats and SARS-CoV-2 in humans are given in the Methods section. These considerations underpinned alteration of selected parameters, which in turn, were informed by published human data. Using rat-derived estimates (Figure1a and b), the model has an estimated ${R_0} = \,\,2.46$ and stabilizes after approximately 724 days at an endemic state (s*,  e*,  i*,  r+*,  l*) = (412.6, 13.9, 19.6, 572.8, 7.1) in which approximately 41.3% of the population is susceptible to reinfection. The only parameter that we vary, ϕ, the rate at which the environment dissipates infectious components, does not play a large role in incidence and prevalence of disease (Figure S1, Supplementary Material). The higher the value of ϕ, the lower each peak of infection. Since this did not substantially affect the model, we have fixed ϕ to the conservative 0.2 for subsequent models.

**Table 1.** Model parameters using transmission estimates obtained predominantly from in vivo rat data.

Parameter	Definition	Model 1	Model 2	Source
β_H_	High risk short-range transmission: % PCR pos (cohabitation)	0.859	0.859	(14)
β_L_	Low risk long-range transmission: % PCR pos (fomite single housing)	0.268	0.268	(14)
δ	1/(duration between first exposure and first positive PCR)	1/3.477		(14)
α	Rate of viral shedding; inverse of PCR	0.102		(14)
ϕ	Rate environment dissipates contaminated agents	0.2, 0.4, 0.6, 0.8		Estimated
ψ	1/duration of recovery period (average number of PCR positive days)	1/2.477		(14)
m	Average duration of immunity if seropositive (mean of days to reinfection)	145.12		(14)
p	Probability of seroconversion in individuals (% seropositive following positive PCR test)	0.703		(14)
e_1_	% reduction of PCR positivity (single dose of vaccine); vaccine effectiveness	-	0.534	(14)
e_2_	% reduction of PCR positivity (two doses of vaccine); vaccine effectiveness	-	0.70, 0.8, 0.9	(35, 36)
v_1_	An individual enters the vaccination compartment following a fixed rate of first vaccination per time t (day)	-	0.0025,0.005,0.01	(37)
v_2_	1/(duration of waiting period between vaccine doses)	-	1/35 days	(38)
m_v_	Average duration of vaccine induced immunity (mean of days to reinfection)	-	145.12	(14)

**Table 2.** Model parameters using translationally adapted (SDAV to COVID-19) estimate ranges. * Indicates that the parameter choice was only used with the duration of infectious period 1/17.

Parameter	Description	Model 1	Model 2	Source
β_H_	High risk short-range transmission: % PCR pos (inoculation or cohabitation)	(0.05*, 0.1, 0.2, 0.4, 0.8)	(0.05*, 0.1, 0.2, 0.4, 0.8)	(41, 42–47)
β_L_	Low risk long-range transmission: % PCR pos (fomite single housing)	(0.01, 0.125, 0.25)	(0.01, 0.125, 0.25)	(34, 48)
δ	Duration between first exposure and first positive PCR	1/(3, 4, 5, 6)	1/(3, 4, 5, 6)	(49–53)
ψ	Duration of infectious period	1/(10, 17)	1/(10, 17)	(54, 55)
m	Average duration of immunity (mean of days to reinfection)	90, 145.12, 180, 365 days	90, 145.12, 180, 365 days	Estimated
m_v_	Average duration of vaccine induced immunity (mean of days to reinfection)	-	90, 145.12, 180, 365 days	Estimated

Compared to SDAV-infected rats, initial spread of SARS-CoV-2 prior to vaccination occurred over a longer period and involved a lower percentage of the population engaged in heterogeneous and complex mixing patterns. To account for this, we repeated the model using an infection duration of 10 days, and a range of estimates for the transmission parameters ${\beta _H}$ and ${\beta _L}$. This produces an estimated ${R_0}$ ranging from 1.05 to 9.27. As expected (Figure S2a, Supplementary Material), the model is driven by high-risk transmission while low-risk transmission resulted in a slight reduction in infections and a slightly flatter curve. For instance, curves with ${\beta _H} = \,\,0.8$ (purple, blue, and orange) follow a similar infection pattern with the lower rate of ${\beta _L}$ transmission pushing the prevalence and incidence curves slightly longer and lower. Immunity following infection (39) or vaccination (40) for SARS-CoV-2 persists for a variable period; thus, rendering a proportion of individuals susceptible to reinfection. In Figure S2b (Supplementary Material), we provide different estimates of immunity duration. Our model shows that the longer infection-induced immunity lasts, the lower and more spread out the peaks of infection remain.

To provide a sensitivity analysis of our model with human translationally alternated analysis, Figure1c,d shows the variability in the compartments. This plot denotes the median at time *t* for each compartment bounded by the 25th and 75th percentiles based on a grid-search of human-adapted parameters in Table 2, which resulted in 320 plausible combinations. Individual prevalence infection compartment curves are shown in Figure S3 (Supplementary Material). While known that ${{\rm{\beta }}_H}$ is a driving force in the model, varying the incubation period, ${\rm{\delta }}$, produced an infection curve that was flatter and elongated as ${\rm{\delta }}$ increased. As expected, when the infectious period ${\rm{\psi }}$ increases from 10 to 17, there are more infections per day with steeper infection peaks. Figure S4 (Supplementary Material) shows an analysis of endemic stability across all parameter choices. A total of 85.6% of parameter choice iterations run reach endemic stability before 5 years with 318 days as the minimum time to endemic stability (${{\rm{\beta }}_H} = \,\,0.8,\,\,{m^ + } = \,\,90,\,\,{\rm{\delta \,\,}} = \,\,3,\,\,{\rm{\psi \,\,}} = \,\,17)$ and a median of 1119 days (IQR = 1017.75).

In humans, variants of concern have emerged, namely Alpha, Beta, Gamma, Delta, and Omicron, with the spread of these variants having a combination of enhanced transmissibility and evasion of immunity elicited by prior variants (56, 41). Bushman et al. (57) found that a rapid increase in infection frequency and the epidemic size was more influenced by increased transmissibility than partial immune escape. To simulate this, we introduce a more transmissible virus by increasing ${{\rm{\beta }}_H}$ and ${{\rm{\beta }}_L}$ and decreasing ${m^ + }$ by 30 days starting at 270 days, repeated every 180 days. Here, we assume an initial literature estimated ${R_0} = \,\,2.5$, we extend the ratio from the rat model and assume ${{\rm{\beta }}_H} = \,\,3.2{{\rm{\beta }}_L}$ with starting values of ${{\rm{\beta }}_H} = \,\,0.216\,\,and\,\,\,\,{{\rm{\beta }}_L} = \,\,0.067$, and we fix ${\rm{\delta \,\,}} = \,\,4\,\,and\,\,{\rm{\psi \,\,}} = \,\,10$ (Figure1e). Each introduction of a more transmissible virus, increasing ${{\rm{\beta }}_H}$ by 0.05 at each step, with decreasing number of days of natural immunity produces a peak in the prevalence of infection with eventual reversal of infected and seropositive populations. We also produced a model with stable immunity duration with increasing transmissibility (Figure1f).

### Model 2: coronaviral transmission after vaccination

**Fig. 2.**
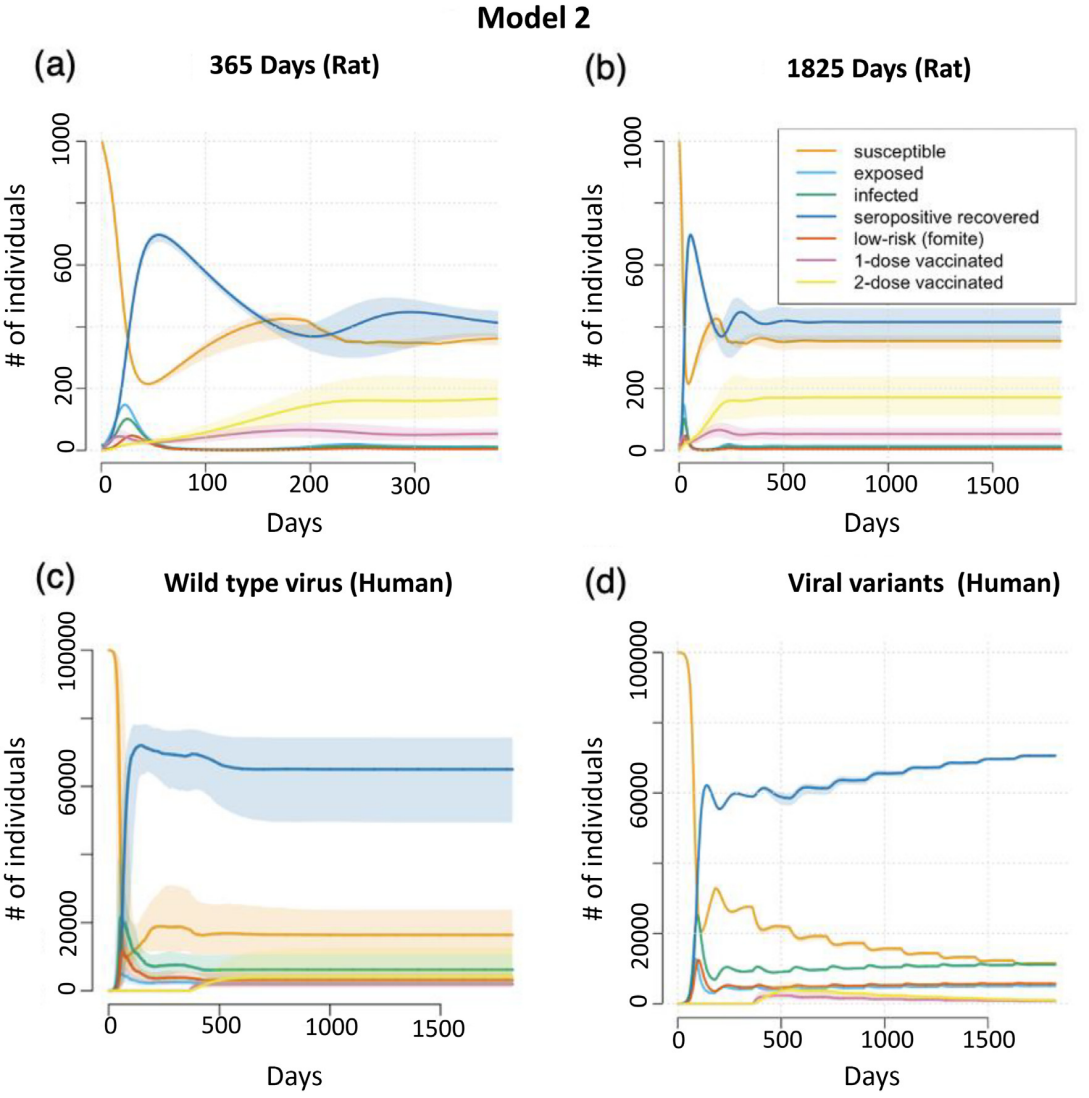
SARS-CoV-2 Disease Compartment Curves for Model 2 with predominantly in vivo rat estimates (a) and (b) and translational human estimates (c) and (d). (a) and (b): curves are the median at time t (25th and 75th percentile) to show the sensitivity of the curves to our set of chosen parameters iterated for 365 days (a) or 1,825 days (b). (c) and (d): curves are the median at time t (25th and 75th percentile) to show the sensitivity of the curves to our set of chosen parameters starting with vaccination at 365 days and iterated for 1,825 days. (c): Model 2 (Human) results using human adapted parameters, with vaccination, and assuming a single ancestral variant. Endemic stability is reached at a median of 1,437 (IQR = 749.25) days. (d): Model 2 (Human), with vaccination introduced after 365 days, each variant increasing in transmissibility every 6 months and remaining parameters held constant. With stable immunity, increasing transmissibility has a smaller and decreasing effect on overall propagation of infection in the population. The compartments and associated curves are denoted in the legend. Population size (closed with no births or deaths) is given as # of individuals on the Y-axis.

We modeled both 1- and 2-dose vaccination strategies using estimates obtained from SARS CoV-2 vaccination reports in humans (37, 38). When these estimates are included in Model 2 using predominantly rat data (Figure2a and b), lower rates of infections over time are observed following introduction of vaccination. The susceptible compartment sharply decreases and only rises slightly due to immune state conferred by vaccination and through natural infection with seropositivity. The cyclic nature of infection is tempered by vaccination with lower peaks of infection across time. Varying parameters in this model, ${v_1},$ vaccination uptake per day and ${e_2}$, rate of reduction in PCR positivity, show that the model is more driven by vaccination uptake and the rate at which the population can be vaccinated than in the efficacy of the vaccine. (Figure S5a and b, Supplementary Material). However, with very low vaccine efficacy at the first and second dose, incidence remains high (Figure S5c, Supplementary Material). Low efficacy for the second vaccination leads to a continued cyclic nature and a longer time to reach an endemic state, whereas high efficacy for the second vaccination produces a steep decline in infections and an endemic state is reached by approximately 600 days as compared to 800 days. With vaccination-induced immunity, long-lasting immunity (${m_v} = \,\,365)$ corresponds to fewer infections per day and sharper decline (Figure S5d, Supplementary Material). Vaccination-induced immunity that spans 60 to 180 days, follow a similar trajectory with few infections per day corresponding to incrementally higher lasting immunity.

Figure2c shows the sensitivity analysis of our translational human estimates with the median per day bounded by the 25th and 75th percentiles (*n* = 8,208 parameter combinations). Retention of the ancestral wild-type virus is assumed throughout, and the rate at which the population is vaccinated, the transmission rates, and duration of immunity greatly affect the model. Endemic stability is reached at a median of 1,437 days (IQR = 749.25) with a minimum of 536 days. A median 15.4% (IQR = 11.9) of the population remains susceptible to reinfection and median infection prevalence is lower than without vaccination (6393.71 (IQR = 6651.4) vs. 7556.655 (IQR = 5655.47)).

In Figure2d, we introduce the vaccine and the variants at specified times throughout the simulation, the vaccine being introduced after 365 days and each variant increasing in transmissibility (${{\rm{\beta }}_H},\,\,{{\rm{\beta }}_L}$) every 6 months. We fixed the remaining parameters similarly. Continued increase in variant transmissibility and immune evasion is the present reality, but is unlikely to continue indefinitely (58). As shown in Figure2d, with stable immunity, increasing transmissibility has a smaller and decreasing effect on overall propagation of infection in the population.

## Discussion

Endemic coronaviral infections in humans and animals are shaped by their tendency to evoke nonsterilizing immunity. Consequently, controlled animal-derived reinfection data can be used to inform transmission models and explore the effects of varying immune duration and transmission following introduction of variants. Using deterministic compartmental models, we used in vivo estimates generated in rats via high- and low-risk transmission routes to model endemic transition through naturally occurring infection alone (Model 1). Based on rat reinfection data, an estimate of 145.12 days for seropositive cases was a starting point for *m*, the average duration of natural immunity. Using rat-derived estimates (Model 1, natural infection), an endemic state achieved by natural infection occurred after approximately 724 days with approximately 41.3% of the population susceptible to reinfection. Addition of heterologous vaccination using RCV (Model 2) attenuated infection peaks. Time to endemic status varied from approximately 600 to 800 days, influenced by vaccine uptake and efficacy. When duration of immunity was varied, incremental accumulation of immunity occurred even with relatively shorter duration of immunity. Immunity lasting less than 365 days resulted in 15% to 20% of the population remaining susceptible to reinfection once the endemic state was reached.

Because our exposure and detection paradigms in rats promoted transmission in the entire population at higher levels than reported in human populations (59, 60), we substituted translationally adapted estimates of SARS-CoV-2 transmission and immune duration in humans in Model 1. As expected, the model was driven by high-risk transmission with low-risk transmission resulting in a slight reduction in infections and a flatter curve. Analysis across all human adapted parameter choices identified a median time to endemic stability of 1,119 days (IQR = 1017.75). The longer infection-induced immunity lasted, the lower and more spread out over time infection peaks were. Our longest modeled duration of immunity (365 days) generated a seasonal pattern similar to that seen with endemic coronaviruses causing seasonal colds (32). We varied infectious period ${\rm{\psi }}$ from 10 to 17 days to accommodate longer reported shedding periods for SARS-CoV-2 than for SDAV and identified correspondingly steeper infection peaks. Iterative analysis across all parameter choices predicted a slightly longer median time to reach endemic stability of 1,437 (IQR = 749.25) days with vaccination, with 15.4% of the population remaining susceptible. This model uses vaccination as an intervention to reduce the chance of infection and reduce transmission (flatten the curve), leading to a median difference of 2.16 (IQR = 2.32) between the basic reproduction number and the effective reproduction number (${R_e}$). However, immunity is modeled as transient and repeated reinfection can occur. Therefore, endemic stability is pushed farther out with a lower % infected at the time of endemic stability for the model with vaccination (Model 2: 6.75% (IQR 6.3%), Model 1: 7.56% (IQR 5.65%)).

Next, we extended human models to include repeated waves of reinfection by introducing a more transmissible virus every 6 months, progressively increasing ${{\rm{\beta }}_H}$ and ${{\rm{\beta }}_L}$. Because successive variants demonstrate partial immune escape (61, 62), we combined each introduction of a more transmissible virus with decreasing number of days of natural immunity. This resulted in reversal of infected and seropositive populations. This scenario is unlikely as increasing transmissibility of variants is likely to stabilize at some point (56), and eventual widespread immunity created by vaccination and successive waves of natural infection is also likely to stabilize (57, 63), leading to the compartmental curves depicted in Figures1f and 2d.

Reinfection rates remain quite high despite combined immunity imparted by vaccination and natural infection vaccination (60). However, our variant scenarios indicate that at endemic equilibrium, vaccination, and natural immunity maintains a much lower infection prevalence compared to an ineffective or absent vaccination. Further, vaccination greatly reduced morbidity and mortality associated with transition to global vaccine and natural infection-induced herd immunity to SARS-CoV-2 (64, 65).

It appears increasingly likely that eventual widespread (but nonsterilizing) immunity combined with reduced viral pathogenicity, as seen with the Omicron variant (66) may reduce SARS-CoV-2 to an endemic seasonal respiratory infection with severe disease manifesting primarily in older or medically vulnerable persons. The observed phenomenon of increased transmissibility, reduced morbidity, and altered tissue tropism demonstrated by the Omicron variant (56, 67) has been previously noted in other species. In pigs, transmissible gastroenteritis virus, an endemic gastrointestinal coronavirus discovered in 1946 (68), underwent a 600 bp deletion in its S gene (69) to generate a new, more transmissible but milder variant with respiratory tropism (porcine respiratory coronavirus) that elicited cross protective immunity to the original virus (10).

Historical precedent for this evolutionary trajectory has been proposed for three of the four globally endemic common cold viruses, HCoV‐229E (70), HCoV‐OC43 (8), and HCoV‐HKU1 (71). HCoV-OC43 and HCoV-HKU1 have been used to model postpandemic SARS-CoV-2 transmission (32) to predict annual seasonal peaks with immune duration of 40 weeks for SARS CoV-2. Duration of immunity to SARS-CoV-2 varies by natural infection (39), disease severity (72), and vaccine type (63), with evidence for cumulative protection achieved by both natural infection and vaccination (63). While repeated natural infection plays an important role in achieving endemic stability, prior immunity is essential to limit morbidity. At endemic stability, seasonal peaks may occur annually (32), but are likely to occur more frequently until that point, driven by new variants, associated variation in duration of immunity and heterogeneity in vaccine uptake, and social distancing practices.

As seen with endemic animal coronaviral infections, a stable endemic state can be interrupted by periodic infection peaks. Widespread host population immunity presents a significant force promoting viral diversification (73), particularly in highly plastic RNA viruses. Infectious bronchitis virus in chickens, first described in 1931, is an endemic coronaviral disease controlled by a broad range of nonsterilizing vaccines that must continually adapt to mutation and recombination (11, 12). Vaccination for IBD elicits evolutionary drive away from vaccine specific antigenic determinants (3), as demonstrated in porcine epidemic diarrhea virus (13). Vaccine evasion by successive SARS-CoV-2 variants is a probable outcome but is unlikely to manifest as complete immune evasion and long-term continued increasing transmissibility of variants (58). Multiple examples in animals illustrate additional means by which the endemic state may be interrupted. These include recombination events between existing coronaviruses (74), emergence of new viruses (7), or reverse zoonotic events (75) in which SARS-CoV-2 adopts a new evolutionary trajectory and then remerge in humans.

Limitations of the study: our models examine infection detectable by a sensitive method (PCR) and do not model morbidity or death, which in humans, is heavily influenced not only by immunity, but by socio-demographics and comorbidity. Additionally, we assumed homogeneous mixing following high and low risk infection routes, compared to heterogeneous mixing in human populations. Our assumption in rat-based models was that a single variant with a given transmissibility remained constant throughout pandemic to epidemic transmission. Therefore, in translationally adapted human models, we introduced variants with increasing transmissibility. Similarly, in rat models, we assumed constant rates of waning immunity and vaccine efficacy throughout the model duration; therefore, a range of duration of immunity was assessed in the human adapted model. Both transmissibility and duration of immunity remain plastic, as the potential remains for new variants with significantly altered transmissibility, tissue tropism, or capacity to caused clinical illness, altering the trajectory of pandemic resolution. Finally, while it was possible to use rat-derived parameter estimates for some parameters, it was not possible for all parameters. Therefore, based on prior convention we used a set of plausible values and ranges based on external resources. However, compartmental models are highly sensitive to parameter choice, and inappropriate parameter choice can lead to bias in model outputs. Therefore, we provide sensitivity analyses of all combinations of our uncertain parameters and discuss the implications on infectious peaks, duration, and endemicity.

## Materials and Methods

### Rat SDAV infection, reinfection, and vaccination studies

Mixing interactions characterizing infectious disease transmission in human populations are heterogeneous, complex, and dynamic (76, 77). Because the full spectrum of these cannot be feasibly modeled in animals, we assessed immune heterogeneity and duration associated with infection and reinfection via high and low risk exposure routes (14). Beginning with a defined SDAV inoculum, we established initial infection using high risk (H: cohabitation) and low risk (L: fomite) transmission to mimic heterogeneous viral exposure in a naturally infected population (14). In the high-risk (H) group, cohabiting animals experienced short-range viral exposure through direct physical contact, aerosol, or droplet routes (34, 78, 79). In the low-risk (L) group, long-range viral exposure via fomites occurred (15, 34, 48). The capacity for previously naturally exposed seropositive animals to shed virus and infect naïve animals after re-exposure was compared to a similar risk from animals exposed to SDAV after heterologous vaccination with Parker's Rat Coronavirus (RCV), a closely related coronavirus (80, 81).

Experimental design and outcomes have been previously published (14) and are briefly summarized here. Young adult Sprague–Dawley rats, evenly split by sex, were infected as follows:

*Initial infection*(*n* = 105): for the cohabitation group, one to three naïve animals were placed with an SDAV-inoculated rat for 24 hours, before being separated from the inoculated rat and placed in a new clean cage. For the fomite group, two to three naïve animals were placed in a contaminated cage that had been previously inhabited by an inoculated rat. After 24 hours, fomite-exposed rats were relocated to a new clean cage in small groups of two to three animals (fomite cohabitation group) or singly (fomite single group). These exposures occurred 48 hours after inoculating the source rat, at a time when viral shedding and upper respiratory tract infection are established (9, 17). To detect viral shedding using semiquantitative RT-PCR, oral swabs were taken on day 2, 3, 4, 7, and 10 postexposure. Seroconversion was assessed 6 weeks after exposure to SDAV. Animal numbers were: inoculated (*n* = 19), cohabitation exposed (*n* = 31), fomite single (*n* = 25), fomite-cohabitation (*n* = 30), and mock inoculated (*n* = 10). Rats were evenly split by sex.

*Reinfection*(*n* = 106): of animals used in the reinfection study, 40 were seronegative and 66 were seropositive. Rats that had originally received intranasal infection with SDAV (inoculated rats) were reinfected intranasally again with the same viral dose (*n* = 18). Naïve seronegative rats (mock inoculated rats from the prior experiment or naïve purchased rats) received intranasal inoculations to provide a source of infection for remaining animals (*n* = 18). Remaining animals (*n* = 22 seronegative rats and *n* = 48 seropositive rats) were randomly assigned direct contact, fomite contact-cohabitation, and fomite contact-singly housed groups for their second exposure. Exposure and testing paradigms were identical to those described for initial reinfection. Time between initial and second exposure ranged from 113 to 165 days. Animals were evenly split by sex and aged 6 to 7 months at sacrifice.

*Vaccination:* RCV has been shown to elicit protective cross-immunity to SDAV (25) and was used in this context as a heterologous vaccine for SDAV. Naïve rats were inoculated intranasally with RCV (*n* = 12), and seroconversion confirmed 1 month later. RCV vaccinated rats were challenged by intranasal inoculation with SDAV 6 weeks later, and viral shedding assessed as described above.

Data used to generate estimates for subsequent modeling are given in Table 3. On initial exposure, 74% of rats shed virus across all exposure groups. Amount and duration of viral shedding and seroconversion following initial natural infection was heterogeneous and influenced by route of exposure. Reinfection of rats that emerged without seroconverting after the initial infection generated similar results. A majority (59%) of naturally infected seropositive rats shed virus on re-exposure after 3.7 to 5.5 months. After vaccination, shedding occurred in a third of animals following SDAV exposure. In both groups, viral shedding occurred at lower levels and for shorter shedding duration following reinfection. Despite reduced viral shedding compared to initial infections, viral shedding by naturally reinfected or vaccinated animals was able to result in transmission to a small proportion of susceptible individuals (14).

**Table 3.** Shedding and seroconversion data obtained from rat experiments used to generate estimates in Table 1.

Exposure type	PCR positive	Cq (mean, range)	Shedding duration (mean, range)	Sero+	Sero−	PCRNeg, Sero+	PCRPos, Sero−
*Initial infection (n* *=* *105)*							
Inoculation (*n* = 19)	19/19 (100%)	31.5; 25.2 to 37.4	3.1; 2 to 4	19 (100%)	0	0	0
Cohabitation (*n* = 31)	31/31 (100%)	31.3; 25.5 to 36.0	2.9; 1 to 3	31 (100%)	0	0	0
Fomite-cohab (*n* = 30)	22/30 (73.3%)	32.9; 27.4 to 36.7	1.5; 1 to 3	15 (50%)	15 (50%)	9 (30%)	9 (30%)
Fomite-single (*n* = 25)	6/25 (24%)	33.2; 28.6 to 36.2	1.5; 1 to 4	2 (8%)	22 (88%)*	1 (5%)	4 (16%)
*Reinfection (seropositive animals; n* *=* *66)*							
Inoculation (*n* = 18)	7/18 (38.9%)	32.7; 28.9 to 36.4	1.7; 1 to 4	–	–	–	–
Cohabitation (*n* = 19)	15/19 (78.9%)	34.1; 29.1 to 36.6	2; 1 to 4	–	–	–	–
Fomite-cohab (*n* = 17)	13/17 (76.5%)	34.4; 30.7 to 37.9	1.7; 1 to 2	–	–	–	–
Fomite-single (*n* = 12)	4/12 (33.3%)	34.5; 33.0 to 36.2	1	–	–	–	–
*Reinfection (seronegative animals; n* *=* *40)*							
Inoculation (*n* = 18)	18/18 (100%)	30.6; 23.5 to 35.3	3; 2 to 4	18 (100%)	0	0	0
Cohabitation (*n* = 9)	8/9 (88.8%)	31.9; 27.3 to 33.4	1.4; 1 to 3	8 (88.8%)	1 (11.1%)	1 (11.1%)	1 (11.1%)
Fomite-cohab (*n* = 9)	6/9 (66.6%)	31.3; 27.2 to 34.5	1.6; 1 to 4	9 (100%)	0	0	0
Fomite-single (*n* = 4)	1/4 (25%)	33.9; 33.9 to 34.0	2; 2	1 (25%)	3 (75%)	1 (25%)	3 (75%)
*Vaccination*							
RCV vaccinated, then SDAV inoculated (*n* = 12)	4/12 (33.3%)	32.5 (30.1 to 34.5)	1.8 (1 to 3)	–	–	–	–

Cq = quantification cycle. Only animals with viral shedding (Cq < 40 cycles) are included in Cq and shedding time calculations. Observation time points comprised postexposure days 2, 3, 4, 7, and 10.

For reinfection experiments, both seropositive and seronegative animals from initial infection were randomized between initial and subsequent routes of infection and were exposed to naïve animals or their cages after these were inoculated with SDAV.

−: Seropositive animals were not retested for seroconversion after reinfection.

* One rat in the fomite single group died of unrelated causes before blood was taken for serology.

### Translational comparisons between transmission of SDAV and SARS-CoV-2

When using these experimental rat-derived estimates to model SARS-CoV-2 transmission in humans, we considered the following differences:

- In contrast to SARS-CoV-2 propagation in human populations, our entire rat population was exposed within a narrow time-period (2 months) during initial infection, followed by re-exposure of the entire population approximately 4 months later.
- Timed exposure and repeated testing using a sensitive method (PCR) after SDAV infection (14) allowed us to detect viral shedding rates on re-exposure that are much higher than those reported in humans (82) in which substantial infection is undetected (83).
- The incubation period between exposure and viral shedding differs slightly between SDAV (approximately 2 days (14)) and SARS-CoV-2 variants with incubation times ranging from 3 to 4 days with Omicron and Delta variants, respectively (84) and 5 to 6 days with prior variants (49, 50). Duration of viral shedding from the respiratory tract is 3 to 10 days in SDAV (14); in COVID-19 this varies from an average of 8 to 10 days in naïve individuals post onset of symptoms (54) to 5.5 days in vaccinated people (85).
- Like SARS-CoV-2 infection (21) or vaccination (22–24), prior SDAV infection or RCV heterologous vaccination (25) do not result in sterilizing immunity. Our assumption was that immunity achieved by RCV is close to that achieved by natural SDAV infection, and that immunity declines at a similar rate (25).
- We modeled transmission only, not disease; thus, modeling viral propagation in healthy often asymptomatic humans, is a common means of SARS-CoV-2 transmission (86). No deaths occurred in our rats; consequently, the population size was assumed to be constant without birth or natural death.

### Compartmental models for infectious disease

Epidemiologic compartmental models divide the population into compartments of disjoint classes, which change within a time *t*, represented by a day in our models (87). We focused on extending the basic SIR (susceptible–infectious–recovered) model using ordinary differential equations (88). We chose to model this deterministically, as opposed to stochastically, due to the sheer volume of cases. The COVID-19 pandemic is the sum of a large number of small individual effects; therefore, the weak law of large numbers diminishes the effects of stochasticity (89). Model assumptions were: (1) no demographic effects, meaning the population is constant, (2) homogeneous mixing, and (3) the population size assumed constant without birth or natural death (89). To mirror SARS-CoV-2 in humans, we added an exposed, E, compartment to the SIR model given an incubation period between a susceptible person becoming infected and shedding the virus (50). In the following sections, we outline the modeling of transmission and immunity and their necessary parameters estimated either by literature or from our in vivo rat studies.

- High risk vs. Low risk transmission

Similarly to SDAV transmission in rats, humans experience high-risk (H) and low-risk (L) exposure (34) to SARS-CoV-2. We modeled fomite transmission similarly to previously engineered compartmental models (48, 90).

In Table 1, we provide estimates for the transmission parameter (β) for each group using predominantly in vivo rat data (14).The short-range high-risk transmission rate β_H_ is the probability of transmission from cohabitation with an infected individual in infected (I) and those in susceptible (S), and the number of contacts per day. This probability, based on our data, was 0.859. The transmission rate β_L_ represents the probability of transmission from the virus in the environment or contaminated surfaces (L) and the susceptible individuals S, and the number of contacts per day. From our data, this was estimated as β_L_ = 0.268. With the SDAV rat model, while in comparison to high-risk transmission, transmission due to fomite exposure followed by solo housing is relatively low but remains a source of transmission risk (14). Therefore, this data estimated α, the rate the virus is shed per day, (i.e. the rate at which infectious rats create contaminated agents) at 0.102 and ϕ, the rate the environment, in this case a cage, dissipates the contaminated agents. We varied the parameter ϕ = (0.2-0.8).

Next, we generated a range of estimates reflecting translational differences between the rat study and SARS-CoV-2 transmission in human populations (Table 2). While fomite transmission can occur (91), the majority of SARS-CoV-2 transmission occurs through close-range exposure, such as in enclosed indoor spaces (92, 93). We varied the parameter β in each exposure group to accommodate transmission rates in humans. This modeled comparably smaller proportions of humans exposed, shorter duration of meaningful contact, and less closely cohabiting human populations at lower values for β, while providing a means to assess the impact of highly transmissible variants at higher values for β. Initial estimates of ${R_0}$, the basic reproduction number, range from 1.4 to 5 with a median of 2.5 (42–45), approximating our rat derived estimate of ${R_0}$ = 2.46. Estimates for ${R_0}$ of the Delta variant range from 3.2 to 8 (42, 45). Transmissibility of the Omicron BA.1 variant is approximately 3.2 times that of Delta (41), with an ${R_0}$ of 8.2 (46). Omicron BA.2 is approximately 1.4 times more transmissible than BA.1 (47). Since ${R_0}$ tends to be derivative of the duration of infectivity, the likelihood of transmission, and the meaningful contact rate, our choices of ${{\rm{\beta }}_H}$ and ${{\rm{\beta }}_L}$ reflect that.

i. Seroconversion

Seroconversion is variable in patients with a PCR positive test with both seropositivity and seronegativity correlating with severity of disease (94, 95). With seronegative cases, we assumed that infections naturally cleared by the individual do not confer protective immunity, reducing our model to a SEIS (susceptible–exposed–infectious–susceptible) model (95). We assumed that seropositive cases confer protective immunity for a period, shifting our model to a SEIRS (susceptible–exposed–infectious–recovered–susceptible). Similarly, the SDAV rat data indicated that a statistically significant association of seroconversion occurred with larger amounts and duration of viral shedding (14). Data from the rat study indicate that in a small proportion of cases, viral shedding detectable by PCR does not always result in seroconversion. Therefore, we estimated our parameter *p*, the rate at which seroconversion occurs, at 77.59%.

ii. Waning immunity and reinfection

Waning immunity and reinfection have been widely reported for SARS-CoV-2 naturally acquired and vaccination immunity (22), and used as markers of epidemic emergence and persistence (95, 96). We defined *m*, the average duration of natural immunity, with an estimate of 145.12 for seropositive cases. Seronegative cases are immediately returned to the susceptible pool. Extrapolating to human populations, we broadened our estimates to include a range reflecting short to long-term duration of immunity (90; 145.12,180; 365 days).

iii. Vaccination

We incorporated our vaccination compartment similarly to previous models (97). An individual enters the vaccination compartment following a fixed rate of first vaccination per time *t* (day), which we varied ${v_1}$ = (0.0025, 0.005, and 0.01) (37). We estimated the duration between vaccine doses to be on average 35 days (37, 38). Depending on vaccine effectiveness, a subset of the vaccinated population can become infected (98). We estimated the % reduction in testing PCR positive for those who were 1-dose vaccinated compared to those who were not vaccinated in our SDAV rat model to be e_1_ = 1−(0.333/0.716) = 0.534. Further reduction in testing PCR positive imparted by subsequent doses is inferred from the literature (35, 36, 99) and estimated as a range e_2_ = (0.7, 0.8, and 0.9). Waning immunity following vaccination, m_v_, is assumed to occur at the same rate as that imparted by natural infection.

iv. Other translationally altered estimates

Because the incubation period for SDAV is short (2 days), the parameter δ (duration between first exposure and first positive PCR) was increased from the observed estimate in the rat study (3.477) to up to 6 days (49–52). Similarly, because viral shedding occurs for a longer period in SARS-CoV-2 patients, the parameter ψ (duration of recovery period) was increased from the observed estimate in the rat study (2.477) to 10 and 17 days (54, 55).

*Model 1: deterministic compartmental model for high risk and low risk transmission, waning immunity, and seroconversion*.

**Fig. 3.**
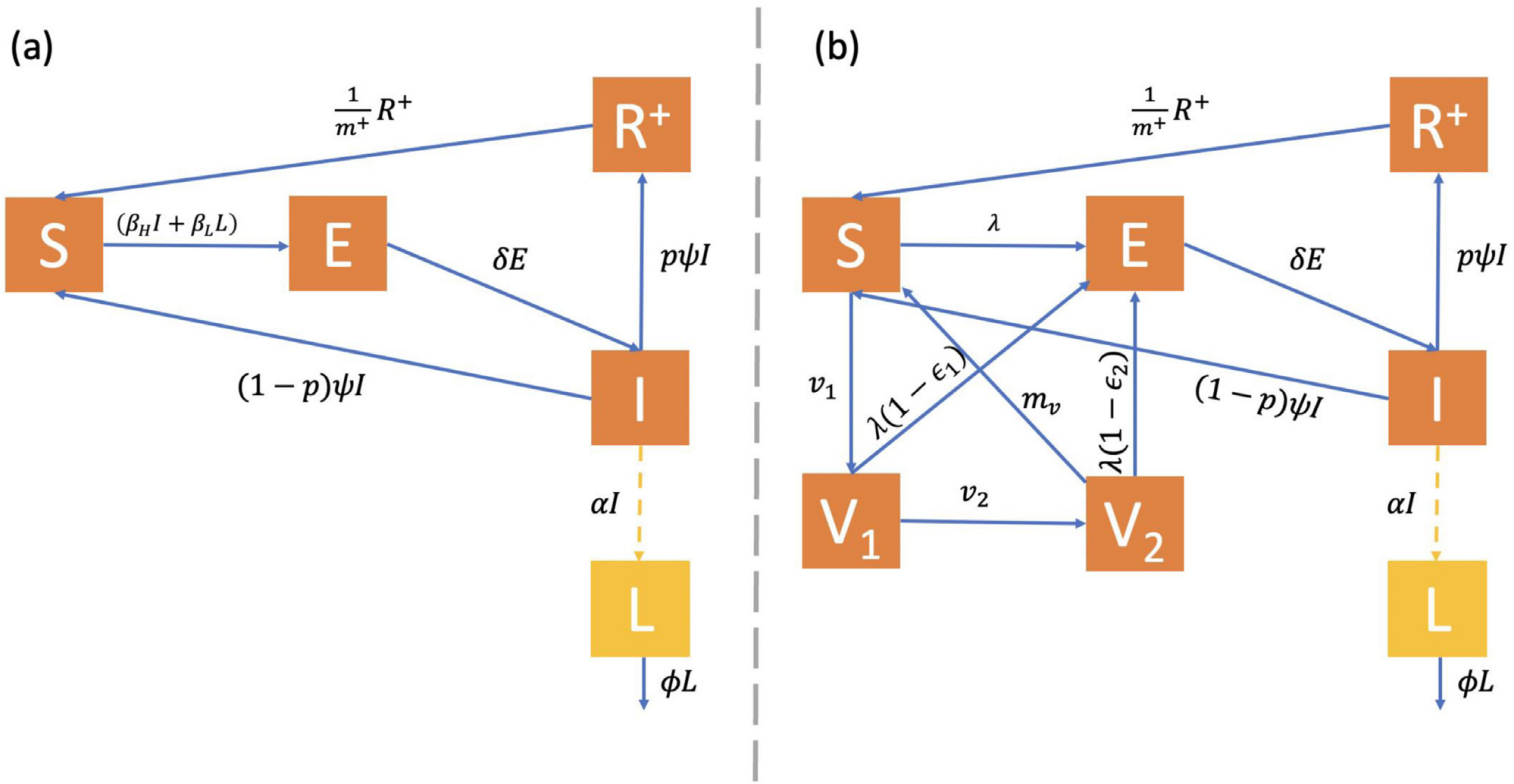
Compartmental model diagram for disease transmission with compartments denoted by susceptible (S), exposed (E), infected (I), seroconverted recovered (R^+^), fomite/low-risk transmission (L), 1-dose vaccination (V_1_), and 2-dose vaccination (V_2_). Arrows indicate the pathway of transmission. Parameters used for disease dynamic estimation with descriptions in Table 1 are labeled above each corresponding arrow. (a) Model 1: deterministic compartmental model for direct and indirect transmission, waning immunity, and seroconversion. (b) Model 2: deterministic compartmental model for direct and indirect transmission, waning immunity, seroconversion, and vaccination. The force of infection, $\lambda \,\, = \frac{{{\beta _H}I + {\beta _L}L}}{N}$.

Our model extends the framework of the SEIR model to incorporate waning immunity, seroconversion, and high- and low-risk transmission (Figure3a). The model consists of five compartments, susceptible (S), exposed (E), infectious (I), recovered (R^+^), and a pseudo-compartment for the amount of virus shed by infected individuals (L). Beginning in the susceptible compartment, individuals can follow the standard pathway to exposed, then infected, and, depending on seroconversion, they either return immediately to the susceptible compartment or move to the recovered compartment. N = S + E + I + R^+^ represents the constant population size. We estimated the rate of duration of exposure from the SDAV rat model as δ = 1/3.477 and the rate of duration of infection as ψ = 1/2.477. We ran the model with initial conditions (*s*(0),  e(0),  *i*(0),  *r+*(0),  *l*(0)) = (1000, 19, 0, 0, 0) based solely on the rat parameters. We increase the susceptible population to 100,000 for the extrapolated human models for a hypothetical scenario of initially naïve population exposed to SARS-CoV-2.

Our model is described in the following equations,

$$\begin{equation*}
{\rm{\,\,}}\frac{{dS}}{{dt}} = \,\, - \frac{{S\left( {{{\rm{\beta }}_H}I + {{\rm{\beta }}_L}L} \right)}}{N} + \frac{1}{{{m^ + }}}{R^ + } + \left( {1 - p} \right){\rm{\psi }}I
\end{equation*}
$$

$$\begin{equation*}
{\rm{\,\,}}\frac{{dE}}{{dt}} = \frac{{S\left( {{{\rm{\beta }}_H}I + {{\rm{\beta }}_L}L} \right)}}{N}\,\, - {\rm{\delta }}E
\end{equation*}
$$

$$\begin{equation*}
{\rm{\,\,}}\frac{{dI}}{{dt}} = \,\,{\rm{\delta }}E - {\rm{\psi }}I
\end{equation*}
$$

$$\begin{equation*}
{\rm{\,\,}}\frac{{d{R^ + }}}{{dt}} = \,\,p{\rm{\psi }}I - \frac{1}{{{m^ + }}}{R^ + }
\end{equation*}
$$

$$\begin{equation*}
{\rm{\,\,}}\frac{{dL}}{{dt}} = \,\,{\rm{\alpha }}I - \phi L
\end{equation*}
$$

The calculation for endemic equilibrium and the reproduction number R_0_ with parameter estimates in Tables 1 and 2${R_0} = \frac{{{{\rm{\beta }}_H}}}{{\rm{\psi }}}\,\, + \frac{{{\rm{\alpha }}{{\rm{\beta }}_L}}}{{{\rm{\psi }}\phi }}$, where the two terms represent infections contributed by high-risk transmission and low risk transmission. For diseases that are endemic, estimating the incidence and prevalence, or number of infections at a given time, and the rate new infections arise helps provide estimates for public health strategies. If ${R_0} = \frac{{{\beta _H}}}{\psi }\,\, + \frac{{\alpha {\beta _L}}}{{\psi \phi }} < 1$ the disease dies out. However, if ${R_0} = \frac{{{\beta _H}}}{\psi }\,\, + \frac{{\alpha {\beta _L}}}{{\psi \phi }} > 1$, the disease remains in the population. Therefore, we discuss the stability of the number of infections given our parameters and model specification.

*Model 2*: *deterministic compartmental model for high and low transmission, waning immunity, seroconversion, and vaccination*.

A vaccination compartment was added to our previous model (Figure3b) with parameters as estimated in Tables 1 and 2. To simplify our equations, we define the force of infection as ${\rm{\lambda \,\,}} = \frac{{{{\rm{\beta }}_H}I + {{\rm{\beta }}_L}L}}{N}\,\,$. Our model incorporating vaccination is described by the following equations:

$$\begin{equation*}
{\rm{\,\,}}\frac{{dS}}{{dt}} = \,\, - {\rm{\lambda }}S - {v_1}S + \frac{1}{{{m^ + }}}{R^ + } + \left( {1 - p} \right)\phi I + \frac{1}{{{m_v}}}{V_2}
\end{equation*}
$$

$$\begin{equation*}
{\rm{\,\,}}\frac{{dE}}{{dt}} = \,\,{\rm{\lambda }}S + {\rm{\lambda }}\left( {1 - {e_1}} \right){V_1} + {\rm{\lambda }}\left( {1 - {e_2}} \right){V_2} - {\rm{\delta }}E
\end{equation*}
$$

$$\begin{equation*}
{\rm{\,\,}}\frac{{dI}}{{dt}} = \,\,{\rm{\delta }}E - {\rm{\psi }}I
\end{equation*}
$$

$$\begin{equation*}
{\rm{\,\,}}\frac{{d{R^ + }}}{{dt}} = \,\,p\phi I - \frac{1}{{{m^ + }}}{R^ + }
\end{equation*}
$$

$$\begin{equation*}
{\rm{\,\,}}\frac{{dL}}{{dt}} = \,\,{\rm{\alpha }}I - \phi L
\end{equation*}
$$

$$\begin{equation*}
{\rm{\,\,}}\frac{{d{V_1}}}{{dt}} = {v_1}\,\,S - \frac{1}{{{v_2}}}{V_1} - {\rm{\lambda }}\left( {1 - {e_1}} \right){V_1}
\end{equation*}
$$

$$\begin{equation*}
{\rm{\,\,}}\frac{{d{V_2}}}{{dt}} = \frac{1}{{{v_2}}}\,\,{V_1} - {\rm{\lambda }}\left( {1 - {e_2}} \right){V_2} - \frac{1}{{{m_v}}}{V_2}
\end{equation*}
$$

The calculation for endemic equilibrium and the effective reproduction number, after vaccination implementation, ${R_e} = \frac{{{\beta _H}}}{\psi }{\rm{\,\,}}( {\frac{{( {1 - {e_1}} ){v_1}{v_2} + ( {1 - {e_2}} ){v_1}{m_v} + 1}}{{{v_1}{v_2} + {v_1}{m_v} + 1}}} ) + \frac{{\alpha {\beta _L}}}{{\psi \phi }}( {\frac{{( {1 - {e_1}} ){v_1}{v_2} + ( {1 - {e_2}} ){v_1}{m_v} + 1}}{{{v_1}{v_2} + {v_1}{m_v} + 1}}} )$, where the two terms represent infections contributed by high-risk transmission and low risk transmission with an additional multiplier given by the vaccination components and disease free equilibrium $DFE( ( {S(0} ),{\rm{\,\,}}L( 0 ),{\rm{\,\,}}E( 0 ),{\rm{\,\,}}I( 0 ),{\rm{\,\,}}{R^ + }( 0 ),{\rm{\,\,}}V\_1( 0 ),{\rm{\,\,}}V\_2( 0 )){\rm{\,\,}} = ( {\frac{N}{{1 + {v_1}{v_2} + {v_1}{m_v}}}} ){\rm{\,\,}},$$0,0,0,0,{\rm{\,\,}}( {\frac{{{v_1}{v_2}N}}{{1 + {v_1}{v_2} + {v_1}{m_v}}}} ),{\rm{\,\,}}( {\frac{{{v_1}{m_v}N}}{{1 + {v_1}{v_2} + {v_1}{m_v}}}} ) ).{\rm{\,\,}}$Our initial starting conditions remain the same as Model 1; for the model run with human parameters, vaccination begins 365 days into the simulation.

## Supplementary Material

pgac096_Supplemental_File

## Data Availability

All details on experimental procedures of rat experiments (including raw transmission data) are publicly available (14). On acceptance, code for models described in this paper will be publicly accessible at https://github.com/sarahmul/COVIDtranslationalepidemics.
