# Supplementary material for: Modeling pandemic to endemic patterns of SARS-CoV-2 transmission using parameters estimated from animal model data
Source: PNAS Nexus. 2022 Jul 1;1(3):pgac096. doi: 10.1093/pnasnexus/pgac096 (PMC9254158; doi:10.1093/pnasnexus/pgac096)
Supplement: pgac096_Supplemental_File [file pgac096_supplemental_file.doc]

**Supplemental Data**


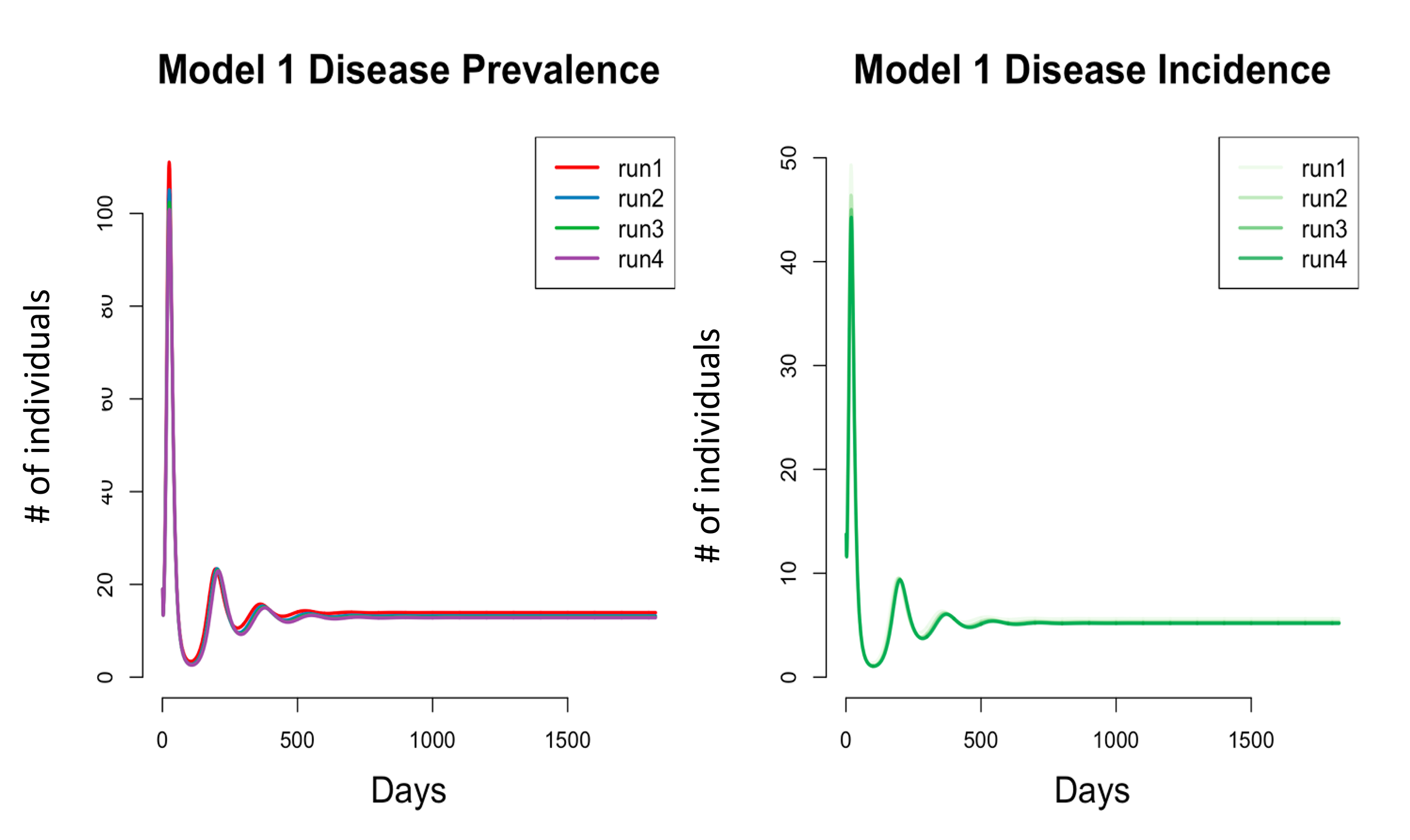


**Figure S1.** Model 1 with rat estimates from **Table 1** showing the sensitivity of the model to the parameter
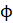
, rate environment dissipates contaminated agents.


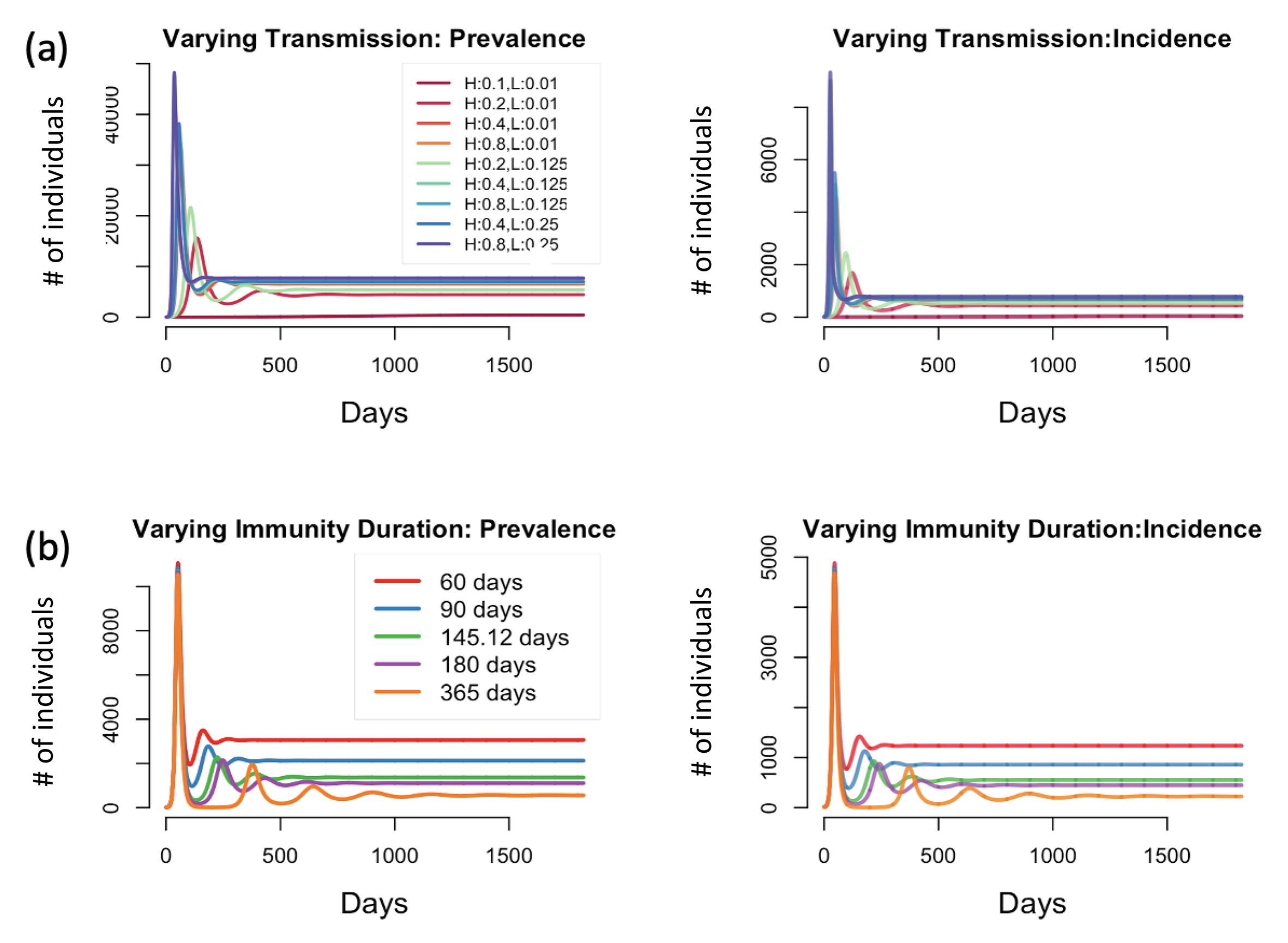


**Figure S2:** Prevalence and Incidence estimates with human-adapted varying transmission and duration of immunity parameters given in Table 2 (a) Varying transmission with H=high-risk transmission,
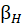
 and L=low-risk transmission,
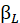
 (b) Varying immunity duration. β_H_ is the primary driving force in the model with high β_H_ resulting in high initial infection rates but more rapid stabilization of infection rates (blue curve: a) compared to lower estimates for β_H_ (red curve: a). Shorter duration of immunity encourages more rapid stabilization of infection rates, but at cost of a greater proportion of the population remaining susceptible to infection (red curve: b) compared to the annual peaks seen with immune duration of 365 days (orange peak: b).


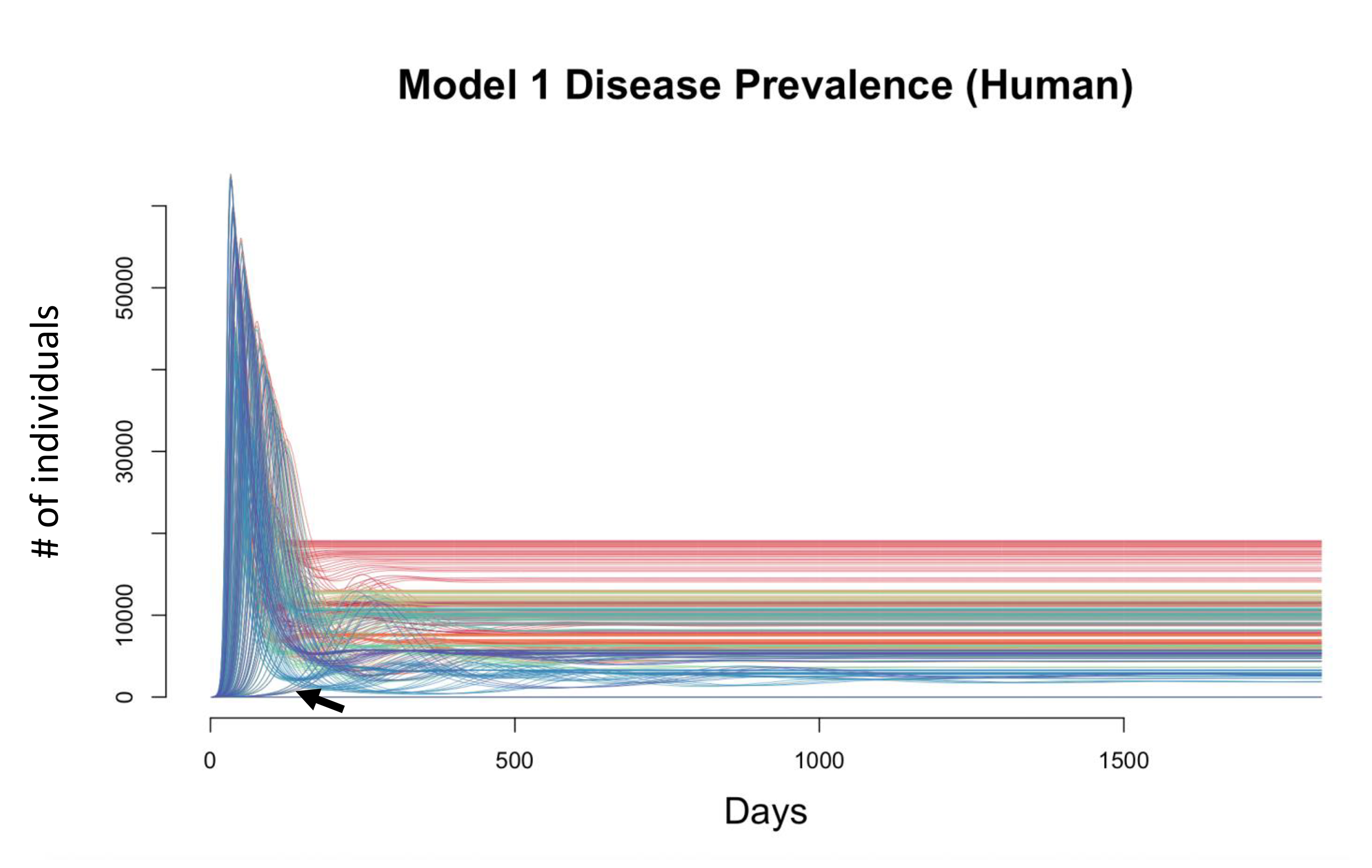


**Figure S3**. Model 1 illustrating individual infection compartment curves derived from 320 plausible combinations obtained with translational human estimates (β_H_, β_L_, ψ, δ and m) given in **Table 2.** Colors of curves correspond to those used in Figure S2. As before, peaks of initial infection and duration of subsequent infection peaks before stabilization are driven primarily by β_H_ Frequency and duration of subsequent infection peaks, and proportion of the susceptible population following endemic equilibrium are largely driven by duration of immunity. Additionally, as ψ increases from 10 to 17, there are more infections per day with steeper infection peaks. Varying the incubation period, δ, produces infection curves that are flatter and elongated as δ increases (arrow).

**
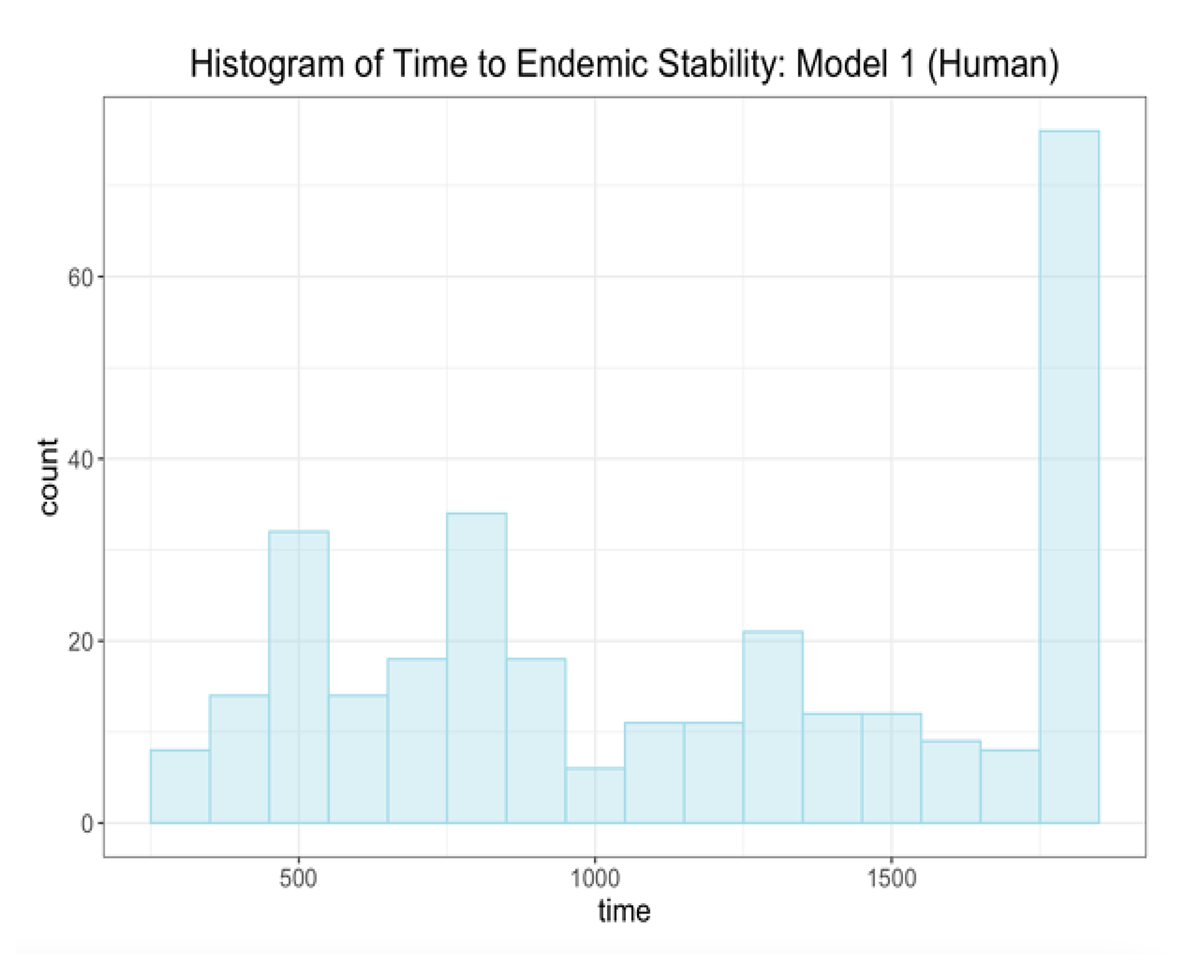
**

(a)

**
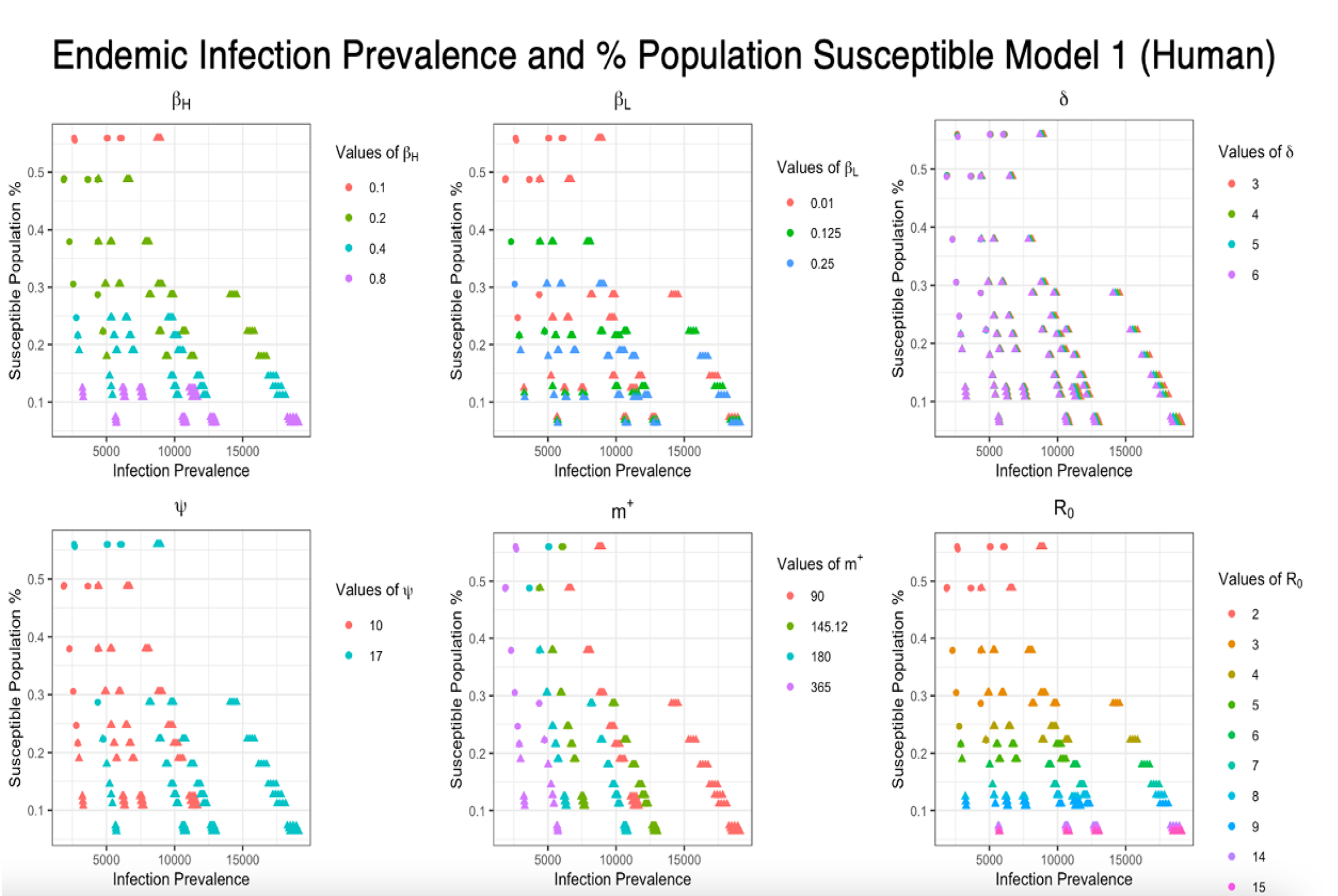
**

(b)

**Figure S4.** Endemicity Stability Analysis of Selected Parameters for Model 1 extrapolated to Humans (a) Histogram of time to endemic stability; 85.6% of sensitivity analysis iterations run reach endemic stability before 5 years with 318 days as the minimum time to endemic stability (
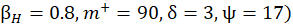
 and a median of 1119 days (IQR=1017.75). (b) Endemic Infection Prevalence and % Population Susceptible scatterplots extrapolated to humans in Model 1. Each scatterplot assigns different colored legends to the varied parameters from Table 2. Lower values of
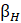
 correspond to higher proportions of the population in the susceptible compartment once stability is reached. Higher values of
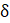
 and
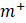
 shift the infection prevalence lower. As
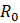
 increases, the percent of the population that is susceptible to reinfection decreases. A 17-day duration of infection compared to a 14-day duration has a higher infection prevalence and a lower susceptible % population. Varying
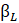
provides minimal distinction across the model iterations.


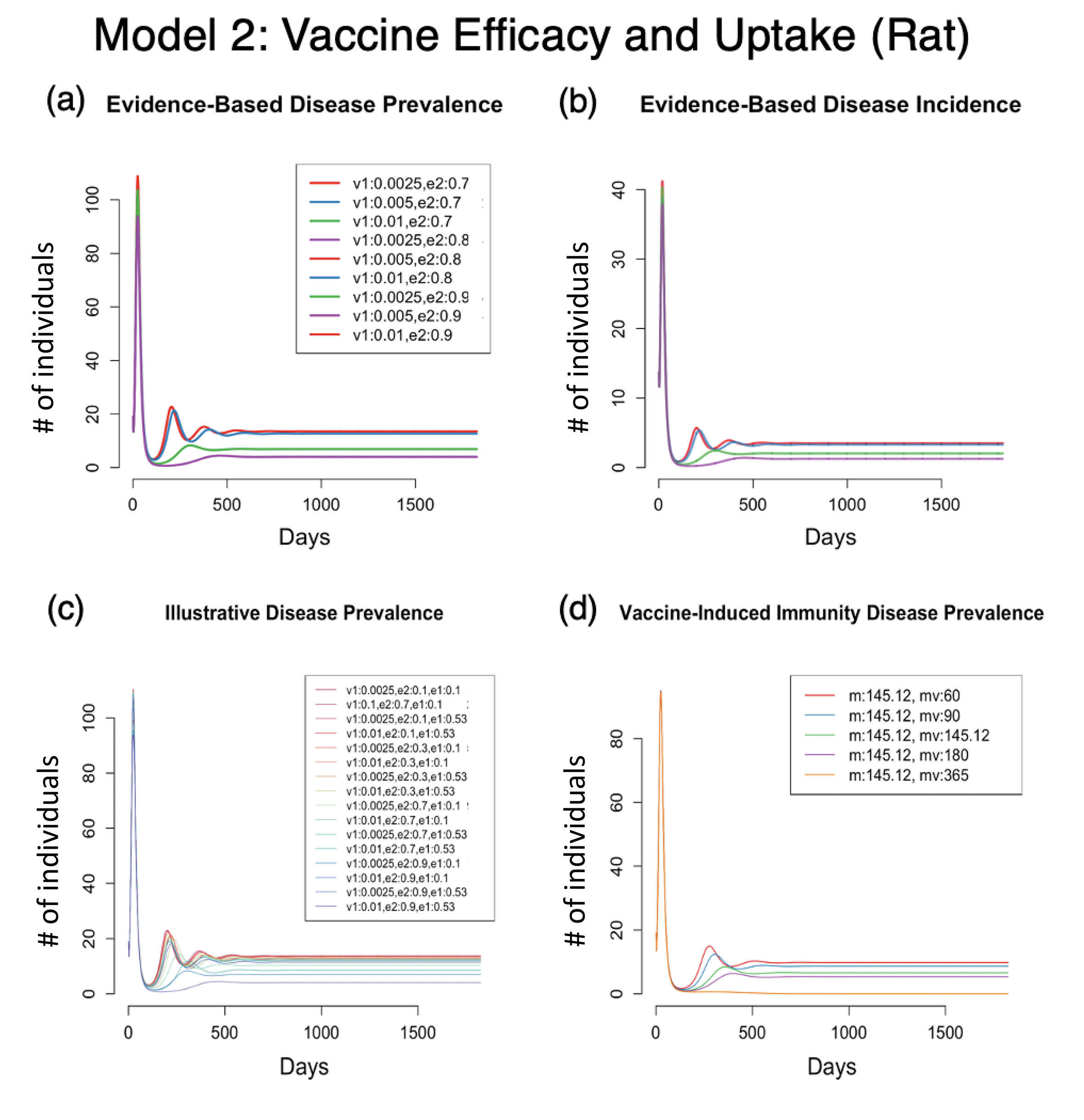


**Figure S5:** Model 2 with varying estimates for vaccine uptake, vaccine efficacy and duration of immunity.

a, b: Using predominantly *in vivo* rat data, prevalence (a) and incidence (b) of infectious individuals after one vaccine dose. Varying parameters in this model,
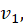
 vaccination uptake per day and
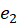
, rate of reduction in PCR positivity, indicate that the model is more driven by vaccination uptake and the rate at which the population can be vaccinated than in the efficacy of the vaccine.

c: Using prevalence of susceptible individuals after two vaccine doses. With very low vaccine efficacy at the first and second dose, incidence remains high. Low efficacy for the second vaccine leads to a continued cyclic nature and a longer time to reach an endemic state, whereas high efficacy for the second vaccine produces a steep decline in infections and an endemic state is reached by approximately 600 days as compared to 800 days.

d: With vaccination-induced immunity, long-lasting immunity (
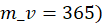
 corresponds to fewer infections per day and sharper decline. Vaccination-induced immunity that spans 60 to 180 days, follow a similar trajectory with few infections per day corresponding to incrementally higher lasting immunity.
